# Mesenchymal stem cell-derived exosomes: a possible therapeutic strategy for repairing heart injuries

**DOI:** 10.3389/fcell.2023.1093113

**Published:** 2023-06-30

**Authors:** Zeshu Zhu, Ping Zhu, Xiongwei Fan, Xiaoyang Mo, Xiushan Wu

**Affiliations:** ^1^ The Center for Heart Development, State Key Laboratory of Development Biology of Freshwater Fish, College of Life Sciences, Hunan Normal University, Changsha, Hunan, China; ^2^ Guangdong Cardiovascular Institute, Guangdong Provincial People’s Hospital, Guangdong Academy of Medical Sciences, Guangzhou, Guangdong, China; ^3^ Guangdong Provincial Key Laboratory of Pathogenesis, Targeted Prevention and Treatment of Heart Disease, Guangzhou, Guangdong, China

**Keywords:** mesenchymal stem cells, exosomes, non-coding RNA, miRNA, heart injury

## Abstract

Mesenchymal stem cells (MSCs) are one of the most potent therapeutic strategies for repairing cardiac injury. It has been shown in the latest studies that MSCs cannot survive in the heart for a long time. Consequently, the exosomes secreted by MSCs may dominate the repair of heart injury and promote the restoration of cardiac cells, vascular proliferation, immune regulation, etc. Based on the current research, the progress of the acting mechanism, application prospects and challenges of exosomes, including non-coding RNA, in repairing cardiac injuries are summarised in this article.

## 1 Introduction

Heart diseases are the leading cause of mortality worldwide and the pathogenesis is insufficient blood supply at the coronary artery. It results in an undersupply of nutrients and oxygen and causes improved fibrosis in the myocardium, causing heart failure and death. Although bypass surgery is the most frequent treatment for heart disease, restoring the blood supply to the cardiac tissue will increase the state of the illness and produce a second injury. Mesenchymal stem cells (MSCs) provide a new way to treat this classical disease.

MSCs are derived from mesoblasts and reside in many organs, such as gum, skeletal muscle, adipose tissue, bone, cardiac and even human umbilical cord blood (Hipp and Atala, 2008; Suzuki et al., 2017; Bagno et al., 2018). MSCs replanted to the injury region will have two effects: 1) sustaining vital cellular processes with differentiation capability and 2) increasing the survivability in a paracrine manner to promote cell activity, induce cell division and inhibit autophagy. However, it has been proven that MSCs cannot stay in the cardiac tissue for long (Muller-Ehmsen et al., 2006; Hu et al., 2018) reported that myocardial cells inhibited MSCs proliferation and differentiation in a paracrine manner. On that basis, MSCs exosomes come into the perspective of researchers as succedaneum.

Mesenchymal stem cell exosomes (MSC-Exos) are bilayer lipid nanovesicles (30–150 nm) derived from MSCs that have been reported to recover injury. For example, Kinnaird et al. reported that an MSC-conditioned medium improved limb function, attenuated incidence and reduced muscle atrophy and fibrosis in mouse hindlimb ischemia (Kinnaird et al., 2004). MSC-Exos enhanced human umbilical vein endothelial cells (HUVEC) to construct tube formation of and decreased infarct size, inflammation response and improved cardiac function of the myocardial infarction (MI) of rats (Teng et al., 2015; Chai et al., 2019) found that MSC-Exos derived from rats notably protected the cardiac from acute IR injury and decreased the fibrotic area, the protein expression of apoptotic and mitochondrial damaged biomarkers in the myocardium. Additionally, MSC-Exos has been reported to possess cardioprotective effects in polymicrobial sepsis and hypoxic pulmonary hypertension (Lee et al., 2012; Wang et al., 2015).

RNA has only been deemed an intermediate product among proteins, DNA and non-coding RNA (NC-RNA). Initially, NC-RNA, which accounting for 98% of the total RNA, was considered a waste product in the process of life metabolism and does not participate in life activities. However, it has been recovered in many studies recent decades that NC-RNA is essential for growth, and the abnormal expression of NC-RNA can also lead to various diseases (Lee et al., 1993; Moss, 2000; Penas and Navarro, 2018; Fung et al., 2019; Tsai and Ogawa, 2019; Dong et al., 2021; Kessler and Schunkert, 2021). There is abundant NC-RNA in MSC-Exos, and researchers have shown that NC-RNA is vital in injury repair. This review aims to introduce recent studies on NC-RNA in MSC-Exos.

## 2 Mesenchymal stem cell exosomes and NC-RNA

MSC-Exos, which are cystic vesicles secreted by MSCs into the environment during physiological activities, composed of lipids, nucleic acids and proteins, are widely involved in the transport of long NC-RNA (lncRNA), microRNA (miRNA), mRNA, proteins and DNA, playing an essential role in inter-cell communication (Sasaki et al., 2019). The composition of MSC-Exos is regulated by MSCs. Nonetheless, MSCs do not possess the specific markers CD105, CD73 and CD90 of MSCs (Anderson et al., 2016), but express specific markers CD9, CD63 and CD81 (Vanhaverbeke et al., 2017). These three markers are commonly used to verify extracted MSC-Exos.

It was shown in many studies that MSC-Exos improve damaged tissue repair. It was confirmed in a study on human umbilical cord stem cell exosomes (hucMSC-Exos) that over 90% of liver tissues absorbed hucMSC-Exos and hucMSC-Exos promoted the repair of CCL4-induced liver injury and inhibited markers of liver fibrosis, including serum HA, ALT and TGF-β1 (Li et al., 2013). The collagen in the liver was reduced after 2 weeks of hucMSC-Exos transplantation and Collagen I and III at the mRNA level decreased significantly after 3 weeks (Li et al., 2013). These are the main results showing that the transplantation of hucMSC-Exos reduces fibrosis in the liver induced by CCl4 (Li et al., 2013). Still, this is not a unique case. Xin Qi et al. found that exosomes secreted from human-induced pluripotent stem cell differentiation-MSCs (e.g., hiPSCs and hiPSC-MSC-Exos) promoted skull defect repair and blood vessel formation in rats (Zou et al., 2019).

Ferguson et al. (2018) showed that NC-RNA in MSC-Exos is essential in this function: the target genes of miRNA, which are included in MSC-Exos, are enriched during cardiovascular generation and relate to the Wnt signal pathway, fibrogenic factor TGF-β and PDGF and proliferation and apoptosis pathways. Interfering the expression of a specific miRNA will affect the repair effect of MSC-Exos on injury, which may be either positive or negative.

## 3 Effect of MSC-Exos miRNA in myocardial injury repair

NC-RNA in MSC-Exos mainly includes miRNA and lncRNA. There are many studies on both miRNAs, and we first introduce them here (Table 1).

**TABLE 1:** Effects of miRNA in MSC-Exos on cardiac injury.

Source of exosome	miRNA	*In vitro*	*In vivo*	Molecular mechanisms	References
Mouse MSCs	miR-125b-5p	Hypoxic and serum deprivation (H/SD) NMCM	MI mouse model	miR-125b-5p in MSC-Exos protects cardiomyocytes by directly inhibiting P53 expression and downregulation Bnip3 expression	Xiao et al. (2018)
Human MSCs	miR-543	CMEC, HEK293T	MI rat model	Human MSC-Exos directly downregulated the expression of COL4A1 by upregulating miR-543 in CMEC and cardiomyocytes, thereby inhibiting the injury caused by myocardial infarction	Yang et al. (2021)
Mouse MSCs	miR-199a-3p	H9C2	DOX induced heart failure (HF) mouse	miR-199a-3p in MSC-SEV activates Akt signaling pathway by upregulating survivin expression in cardiomyocytes, thereby protecting DOX induced cardiomyocytes	Lee et al. (2021)
ADMSCs	miR-671	OGD—treatment of mouse cardiomyocytes CP-M073	MI mouse model	miR-671 in ADMSC-EXOs protect cardiac function in myocardial infarction mice by directly inhibiting TGFBR2 and inhibiting apoptosis related factors as well as inflammatory factors IL-6 and TNF-α	Wang et al. (2021a)
Human EnMSCs	miR-21-5p	Hypoxic treatment of HUVECs and neonatal rat cardiomyocytes (NRCM)	MI mouse model	miR-21-5p in human EnMSC-Exos activates the Akt signaling pathway by directly and negatively regulating PTEN and inhibits cardiac dysfunction caused by MI	Wang et al. (2017)
hucMSCs	miR-1246	OGD—treatment of H9C2 and HUVECs	HF rat model	miR-1246 in hucMSC-Exos antagonizes the damage caused by LAD ligation by directly inhibiting PRSS23 expression	Wang et al. (2021b)
hucMSCs	miR-125b-5p	Hypoxic treatment of H9C2	MI rat model	miR-125b-5p in hucMSC-Exos protect the heart of myocardial infarction rats by directly downregulation Smad7 expression	Wang et al. (2018)
Mouse MSCs	miR-21a-5p	H/SD H9C2; miR-21a knockout neonatal mouse cardiomyocytes (NMCM)	I/R mouse model	BMSC-Exos protects myocardial cells by inhibiting PDCD4, PTEN, FasL and Peli1 by conduction mir-21a-5P to cardiomyocytes in myocardial infarction region	Luther et al. (2018)
BMSCs	miR-486-5p	H/R treatment of H9C2	I/R rat model	miR-486-5p in BMSC-EXOs activates PTEN/PI3K/AKT signaling pathway by directly downregulation PTEN in cardiomyocytes	Sun et al. (2019)
ADSC	miR-221/miR-222	H2O2 treatment of H9C2	I/R mouse model	Mir-221/Mir-222 in ADSC-Exo inhibits ischemia-reperfusion injury of the mouse heart by inhibiting PUMA and ETS-1 through conserved sequences at the 5′UTR	Lai et al. (2020)
MSCs	miR-21-5p	LPS treatment of RAW264.7	I/R mouse model	miR-21-5p in MSC-EXOs is important in inducing polarize M1 macrophages to M2 macrophages	Shen and He (2021)
BMSCs	miRNA-182	RAW264.7	I/R mouse model	miR-182 in BMSC-Exos promote PI3K/Akt signaling pathway by inhibiting TLR4 in macrophages. It downregulates the markers of M1 (IL-6, TNFα, IL-1β, and iNOS) and upregulates the markers of M2 (Arg1, TGFβ, CD206 and IL-10), promotes that polarizing macrophages M1 to M2, and alleviates the damage of inflammating to the heart	Zhao et al. (2019a)

### 3.1 Protective effect of miRNA in cardiac injury

**FIGURE 1:**
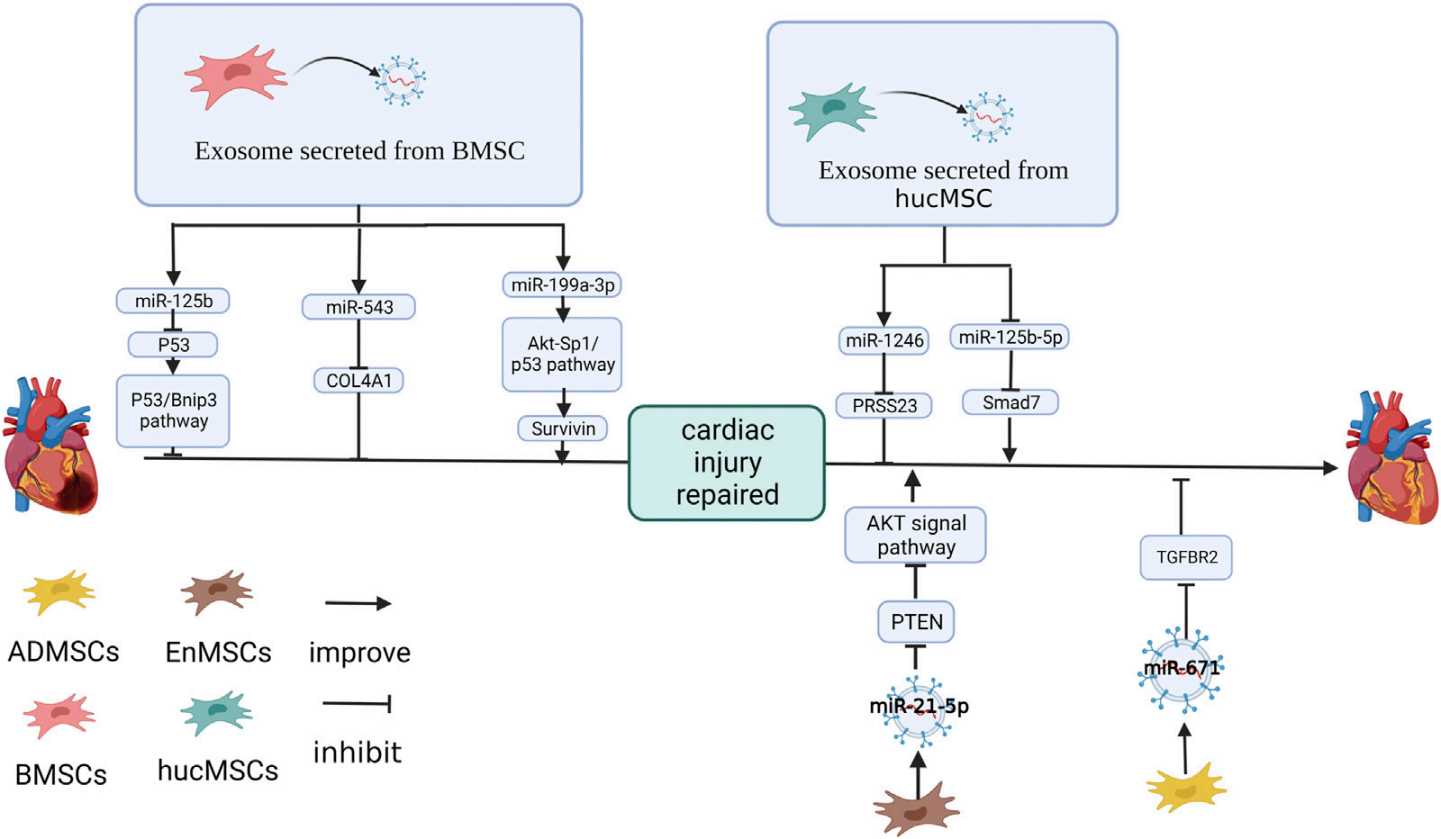
Effect and mechanism of miRNA on MSC-Exos repair myocardial infarction: miR-125b (Xiao et al., 2018), miR-543 (Yang et al., 2021) in BMSC-Exos. miR-1246 (Wang et al., 2021b) in hucMSC-Exos and miR-21-5p (Wang et al., 2017) in EnMSC-Exos. miR-671 in ADMSC-Exos inhibits the expression of target genes (Wang et al., 2021a). miR-199a-3p in BMSC-Exos improves the expression of target genes (Lee et al., 2021). hucMSC-Exos inhibits miR-125b-5p in cardiomyocytes (Wang et al., 2018). The figure was created with BioRender.com.

MSCs can be divided into umbilical cord mesenchymal stem cells, bone marrow mesenchymal stem cells (BMSCs), adipose mesenchymal stem cells (ADMSCs), etc. Among these, BMSCs are the most studied. If the source site of MSCs is not marked in the original text, bone marrow is the default site used in this review. Here we introduce miRNA in bone marrow MSCs exosomes (BMSC-Exos) in heart injury (Figure 1).

miR-125b-5p was upregulated in cardiomyocyte after MSC-Exos treatment. Knockdown of miR-125b in MSCs downregulates miR-125b-5p in MSC-Exos, impaired the protection of MSC-Exos on serum oxygen stripping cardiomyocyte and increased autophagy flux (Xiao et al., 2018). It has been shown in signaling pathway studies that B-cell lymphoma 2-interacting protein 3 (Bnip3) and p53 protein in cardiomyocytes are affected by miR-125b (Xiao et al., 2018). Compared with the control group, the cardiac autophagy flux and infarct size of the mice with MI was increased when treated with miR-125b-5p inhibiter pretreating MSC-Exos. Western blotting results showed in the p53 protein being the target gene of miR-125b-5p (Xiao et al., 2018).

Human MSC-Exos can improve the angiogenesis, proliferation and migration of cardiac microvascular endothelial cells (CMECs), inhibiting miR-543 limits this effect (Yang et al., 2021). Upregulating miR-543 in HEK293T cells inhibited COL4A1 at the protein level, and the predicted site mutation of COL4A1 broke this link. *In vivo*, human MSC-Exos significantly reduced the degree of myocardial necrosis, neutrophil infiltration and myocardial injury in rats with MI, promoted the proliferation of cardiomyocytes, upregulated miR-543 and inhibited COL4A1 (Yang et al., 2021).

MSC-Exos upregulate Sp1 and inhibit p53 through Akt activation. Therefore, survivin, a therapeutic target for doxorubicin (DOX)-induced cardiomyopathy, is upregulated. The inhibitory effect of MSC-Exos on cell apoptosis is reduced by the inhibition of survivin expression (Lee et al., 2014; Lee et al., 2021; Lee et al., 2021) showed that three miRNAs, miR-424-5p, miR-21-5p and miR-199a-3p, had high expression levels in MSC-SEV and activated the Akt signaling pathway. miR-199a-3p was the only one that significantly upregulated survivin expression in H9C2 cells. Inhibiting miR-199a-3p in DOX-induced cardiomyocytes effectively reversed the effects of MSC-Exos, downregulating survivin (Lee et al., 2021). In another study, Fan et al. found that fibrotic cardiomyocyte autophagy was also promoted by miR-199a-3p in BMSC-Exos by inhibiting mTOR, thereby reducing cardiac fibrosis (Fan et al., 2022).

### 3.2 The miRNA from MSC-Exos of different species on cardiac function

Currently, the exosomes used in previous studies were mainly extracted from BMSCs. However, this does not mean that MSCs from other sources have lost their research value. For example, among umbilical cord blood MSCs (UCBMSCs), BMSCs and ADMSCs, ADMSCs had the best protective effect on rats with MI (Xu et al., 2020). It has been found in many studies that miRNAs in MSC-Exos from different organs were also different. Here, the role of miRNAs in MSC-Exos from various sources is shown (Figure 1).

Wang et al. (2021a) found that ADMSC-Exos protected cardiomyocytes under an oxygen and glucose deprivation (OGD) environment and inhibited the expression of apoptosis marker factors. It was shown by miRNA microarray analysis results that ADMSC-Exos upregulated miR-671 in cardiomyocytes, and mir-671 was enriched in ADMSC-Exos (Wang et al., 2021a). The inhibition of miR-671 expression caused ADMSC-Exos to lose their antagonistic effect on cardiomyocyte apoptosis, apoptotic factors, and releasing pro-inflammatory factors from cardiomyocytes (Wang et al., 2021a). The action of the miR-671 inhibitor and was stopped and apoptosis-related and inflammatory factors (IL-6, TNF-α) was inhibited by si-TGFBR2 (Wang et al., 2021a). In mice with MI, ADMSC-Exos upregulated miR-671, reduced TGFBR2 and P-SMad2 at the protein level, alleviated the inflammatory response of mouse myocardial tissue, reduced myocardial fibrosis and inhibited the apoptosis of myocardial tissue and these functions were attenuated in miR-671-inhibited ADMSC-Exos (Wang et al., 2021a).

Wang et al. (2017) found that compared with BMSC-Exos and ADMSC-Exos, the endometrium-derived MSCs exosome (EnMSC-Exos) was the best protection of the heart in the MI of rat models. The degree of apoptosis, cardiac function and infarct size in MI rats treated with EnMSC-Exos improved significantly. It has been shown that miRNA miR-21-5p had high expression in EnMSC-Exos (Wang et al., 2017). PTEN, the miR-21-5p target gene, was downregulated in EnMSC-Exos co-cultured NRCM and HUVEC. At the same time, the protein phosphorylation level of Akt and Bcl-2 increased. These functions were inhibited by the silencing of miR-21-5p in EnMSC-Exos (Wang et al., 2017).

Wang et al. (2021b) discovered that hucMSC-Exos improved myocardial angiogenesis of heart failure (HF) in rats, reduced the damage of inflammatory cell infiltration, inhibited apoptosis of OGD-treated cardiomyocytes, caspase-3 activation and promoted tubular structure formation of OGD-treated HUVEC. miR-1246 was highly expressed in hucMSC-Exo, while miR-1246 was significantly decreased in the LAD rat myocardium, OGD-treatment cardiomyocytes and HUVEC (Wang et al., 2021b). It was confirmed that miR-1246 in hucMSC-Exos was absorbed by the recipient cell and downregulating miR-1246 inhibited the effect of hucMSC-Exo on cardiomyocyte and HUVEC (Wang et al., 2021b). HF upregulated Snail and α SMA, and hucMSC-Exo inhibited α-SMA and Snail. In contrast, the downregulation of miR-1246 limited α-SMA and Snail in hucMSC-Exo (Wang et al., 2021b). It was revealed by the detection of miR-1246 target sites that PRSS23 was the target gene of miR-1246, resulting in the upregulation of miR-1246 and thereby inhibiting PRSS23 (Wang et al., 2021b).

Wang et al. (2018) found that hucMSC-Exo overexpressed Smad7 in MI rats. It was shown in a bioassay result that the expression of three NC-RNA, miR-92a-3p, miR-21-5p and miR-125b-5p, was changed after injection or co-culture of hucMSC-Exo and only miR-125b-5p was consistent with *in vitro* and vivo (Wang et al., 2018). Overexpressing miR-125b-5p downregulated Smad7, while mutations in the predicted target at Smad7 3’UTR invalidated this negative regulation (Wang et al., 2018). It has been suggested that hucMSC-Exo upregulates Smad7 by downregulating miR-125b-5p in cardiomyocytes, protecting cardiac tissue from MI (Wang et al., 2018).

### 3.3 The protection of miRNA from MSC-Exos on ischemia/reperfusion (I/R) cardiac

**FIGURE 2:**
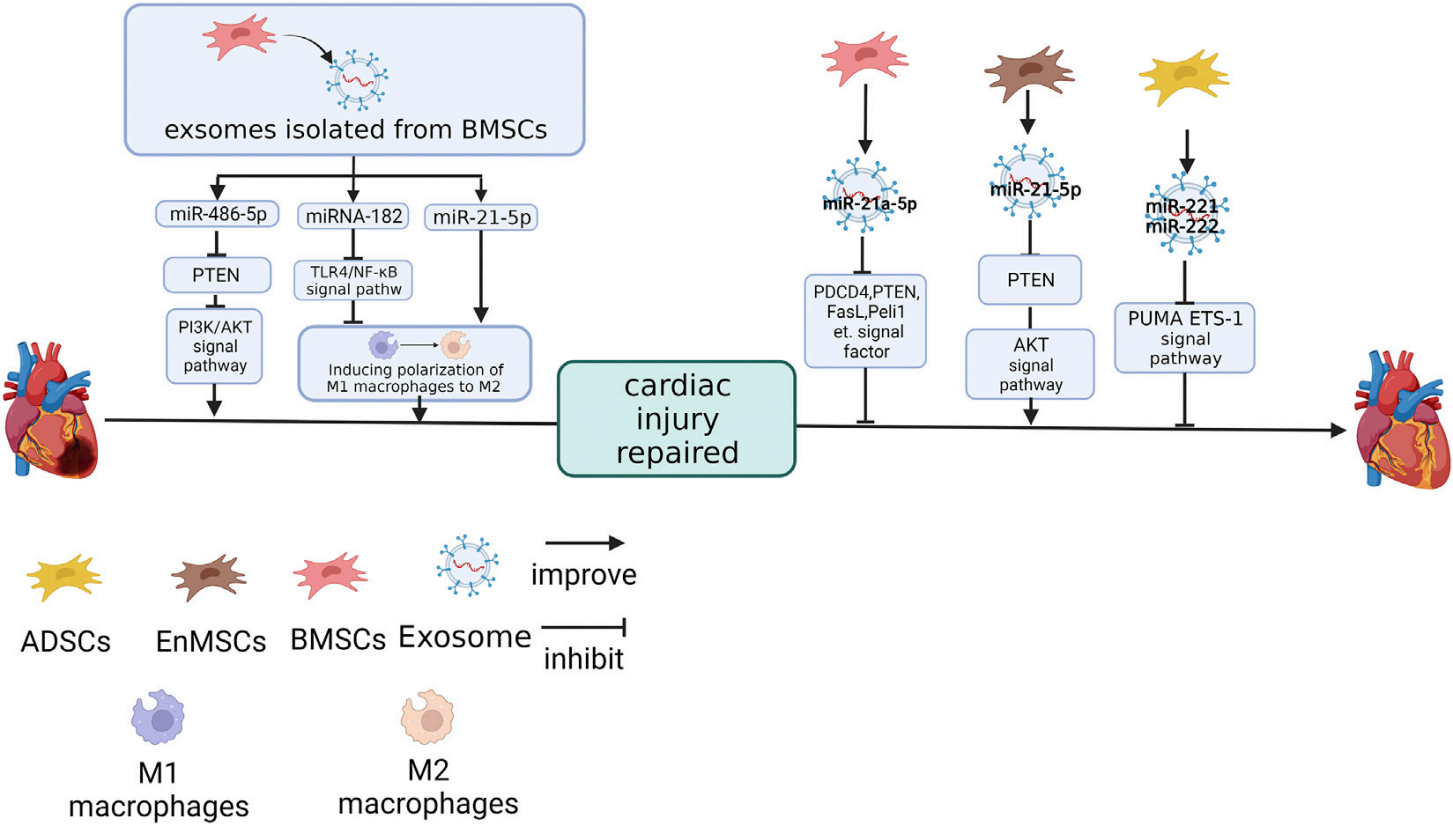
The effect of miRNAs derived from different MSC sources on cardiac I/R injury repair. miR-486-5p and miR-21a-5p (Luther et al., 2018) in BMSC-Exos (Sun et al., 2019). miR-21-5p in EnMSC-Exos (35). miR-221 and miR-222 in ADSC-Exo inhibit the expression of target genes (Lai et al., 2020). miRNA-182 and miR-21-5p in BMSC-Exos promote cardiac function recovery by inducing macrophage polarisation from M1 to M2 (Zhao et al., 2019a; Shen and He, 2021). The figure was created with BioRender.com.

Restoring the blood supply as soon as possible is the most effective way to control injury in acute coronary syndrome. However, it has been shown in many studies that reperfusion may lead to subsequent injury to the ischemic area, which is called ischemia/reperfusion (I/R) injury (Wu et al., 2018). miRNA repairs this damage in many different ways (Figure 2).

Knockdown of miR-21a-5p in MSC-Exos reversed the MSC-Exos effect on the heart of I/R mice, upregulated the protein expressions of PDCD4, PTEN, FasL, Peli1 and cleaved caspase-3, which caused an expansion in the MI area (Luther et al., 2018). The targets of miR-21a-5p were PDCD4, PTEN, FasL and Peli1. These four genes were simultaneously and negatively regulated by miR-21a-5p, suggesting that cell apoptosis was regulated by miR-21a-5p through these four signaling factors (Luther et al., 2018).

It has been pointed out in previous studies that miR-486-5p, which was enriched in the BMSC-Exos, can reduce I/R injury (Baglio et al., 2015; Vinas et al., 2016; Sun et al., 2017; Zhang et al., 2018). Overexpressing miR-486-5p in BMSC-Exos could improve the cell viability and the expression of Bax, downregulate Bcl-2 and PTEN in H9C2 after hypoxia/reoxygenation (H/R) treatment. However, the point mutation in the target site located in the 3’UTR of PTEN eliminates this link. PI3K-p110a and p-Akt, which play a negative regulatory role in PTEN, are positively regulated by miR-486-5p. Cardiac function of I/R rats was also significantly improved when cells were treated with overexpression of miR-486-5p BMSC-Exos (Sun et al., 2019).

Lai et al. (2020) found that miR-222/miR-221 were inhibited after I/R treatment in mouse hearts. However, ADSC-Exo would upregulate miR-222/miR-221 in the I/R mice cardiac tissue, and inhibition of miR-221/miR-222 nullified the protective effect of ADSC-Exo in mouse hearts. This suggests that miR-221/miR-222 are essential for the protective function of exosomes against I/R injury. The upregulation of miR-221/miR-222 inhibited PUMA (a p53-upregulated modulator of apoptosis) and ETS-1 (ETS protooncogene 1) and this association was eliminated by mutations in the predicted PUMA and ETS-1 sites at the 3’UTR.

Interestingly, although MSC-Exo inhibits the infiltration of I/R injured hearts by inflammatory factors, this effect is not achieved by reducing macrophages. Although mouse BMSC-Exos significantly reduce the infiltration of inflammatory factor IL-6 and inflammatory cells in the I/R heart tissue, the removal of macrophages in mice will reduce the efficacy of BMSC-Exos and interfere with the recovery of exosomes to cardiac function in I/R mice and macrophage numbers did not change significantly in I/R mice treated with BMSC-Exos. This indicates that macrophages are necessary for the protective effect of exosomes on cardiac function in mice (Zhao et al., 2019a). Although the number of macrophages was unchanged, the expression of TGFb, CD206, Arg1 and IL-10 in M2 macrophages was upregulated in BMSC-Exos-treated I/R mice. This suggests that BMSC-Exos transformed macrophages away from the M1 phenotype into M2 and this phenomenon was also repeated in the co-culture of RAW264.7 and BMSC-Exos (Zhao et al., 2019a).

NC-RNA is also essential to this effect. miR-182 in BMSC-Exos reproduces the impact of BMSC-Exos, induce M1 macrophages to polarise to M2, on macrophages *in vitro*. In the other hand, BMSC-Exos treated with miR-182-inhibitor downregulates M1 markers (e.g., IL-1β, IL-6, TNFα, iNOS) and upregulates M2 markers (e.g., CD206, TGFβ, IL-10 and Arg1) after being internalised by macrophages (Zhao et al., 2019a). miR-182 inhibits the expression of TLR4 in macrophages at the protein level without changing TLR4 at the mRNA level. Inhibition of TLR4 can activate PI3K/Akt signaling pathway, which is vital in transforming inflammatory M2 macrophages (Zhao et al., 2019a). TLR4 knockout mice had similar phenotypes after I/R treatment, such as the recovery of heart function and increased M2 macrophage proportion in BMSC-Exos-treated mice (Zhao et al., 2019a).

miR-182 is not alone. Inhibiting miR-21-5p in MSC-Exos is not only effect on cardiomyocytes, but also promoted macrophages to polarise with the M1 phenotype and improved the inflammatory response (Shen and He, 2021). The miR-21-5p mimic promoted the polarisation of macrophages to M2 and reduced inflammatory factors (Shen and He, 2021).

## 4 The effects of culture methods on MSC-Exos and NC-RNA

MSC-Exos promotes injury repair and changes in the culture environment can affect this function. Suxiao Jiuxin, a coronary syndrome medicine, is co-cultured with cardiac-derived mesenchymal stem cells (C-MSCs) and promotes the secretion of C-MSC exosomes through a GTPase-dependent signaling pathway (Ruan et al., 2018a). This exosome increased histone three lysine 27 trimethylation (H3K27 me3), a critical cardiac transcriptional repressor epigenetic chromatin marker, by upregulating histone methylases (e.g., EED, EZH2, and EZH1) and inhibited demethylase (UTX and JMJD3), cell apoptosis and promoted cell proliferation, thereby protecting against the cardiac function injury caused by MI (Ruan et al., 2018b). It has been confirmed in several studies that changing the environment of MSC cultures also alters the expression of NC-RNA (NC-RNA). Here, we discuss the change in NC-RNA in MSC-Exos by pretreatment, the effect of this change on the repair of cardiac injury and the related mechanisms. A summary is provided in Table 2.

**TABLE 2:** The effects of culture methods on miRNA expression in MSC-Exos.

Source of exosome	Spices	Treatment	miRNA	*In vitro*	*In vivo*	Mechanism	References
BMSCs	Human	Treatment 0.5 mg/L lipopolysacc-haride	miR-181-5p	H2O2 treatment H9C2		Mir-181-5p directly acts on ATF2 and inhibits the oxidative stress of H2O2-induce-cardiomyocytes by downregulating ATF2	Liu et al. (2020)
MSCs	Mouse	Pretreatment with 10 µm heme for 24 h	miR-183-5p	Treatment of NMCM with mitochondrial fission activator FCCP	MI mice	Pretreatment with Hemin promote miR-183-5p in exosomes, and then inhibit P-DRP1/Drp1, HMGB1 and p-ERK/ERK in cardiomyocytes, HMGB1 is the target gene of miR-183-5p	Zheng et al. (2021)
BMSCs	Mouse	Treatment with hypoxia (1% O2)	miR-125b-5p	H9C2 of treatment with hypoxia	MI mice	Knockdown miR-125b attenuates the protective effect of Hypo-BMSC-Exos on cardiomyocytes, and upregulates p53 and BAK1 in cardiomyocytes	Zhu et al. (2018b)
BMSCs	Rat	Treatment with hypoxia	miR-24	H9C2 treated with hypoxia	MI rat	Hypo-BMSC-Exos treatment will upregulate miR-24 in cardiomyocytes, and then inhibit Bax, caspase-3, and cleaved caspase-3	Zhang et al. (2019)
BMSCs	Rat	Treatment with hypoxia	miR-98-5p		MI rat	Hypo-BMSC-Exos treatment will upregulate miR-98-5p in cardiomyocytes, directly inhibit TLR4, and activate PI3K/Akt signaling pathway	Zhang et al. (2021)
BMSCs	Mice	Treatment with hypoxia	miR-210	H2O2-induced-H9C2/HUVECs	MI mice	Hypoxia activates HIF-1α and then upregulates nSMase2, thereby increasing miR-210 in Hypo-BMSC-Exos and promoting the protection of Hypo-BMSC-Exos on cardiomyocytes and blood vessel	Zhu et al. (2018a)
BMSCs	Mice	Treatment with H/R	miR-29c	Neonatal rat cardiomyocytes (NRCM) treated with H/R	I/R mouse	H/R treatment decrease miR-29c in BMSC-Exos, and upregulate LC3 and P62 in cultured rat cardiomyocytes, which decreased BMSC-Exos protective effect on cardiomyocytes	Li et al. (2020a)
BMSCs	Rat	Overexpression GATA4	miR-19a and miR-451	Hypoxia treatment NMCM	MI rat	MiR-19a and miR-451 in Exo^gata4^ was upregulated, and the knockdown of miR-19a would eliminate the protective effect of Exo^gata4^ on cardiomyocytes, which was caused by the upregulation of PTEN and BCL, the direct target sites of miR-19a, in cardiomyocytes	Yu et al. (2015)
BMSCs	Rat	Overexpression miR-30e	miR-30e	OGD treatment H9C2	MI mouse	MiR-30e-BMSC-Exos protects cardiomyocytes by blocking NF-κB p65/caspase-9 via inhibiting LOX1 in cardiomyocytes	Pu et al. (2021)
BMSCs	Rat	Overexpression miR-133a	miR-133a	Coxsackie B3 virus treatment NMCM	Coxsackie B3 virus induces viral myocarditis rat	Overexpression of miR-133a in BMSC-Exos will inhibit MAML1 (mastermind-like 1) in cardiomyocytes and inhibit the damage of cardiomyocytes to viral myocarditis caused by Coxsackie B3 virus	Li et al. (2021b)
ADSCs	Rat	Overexpression miR-146a	miR-146a	Overexpression 146a/EGR1 H9c2	MI rat	MiR-146a-exosome decreases apoptosis-related protein caspase-3 and Bax, fibrosis-related protein Collagen I and α-SMA in cardiomyocytes by inhibiting EGR1/TLR4/NFκB signaling pathway, thereby protecting cardiac function	Pan et al. (2019a)
BMSCs	Mouse	Overexpression miR-150-5p	miR-150-5p		MI mouse	MiR-150-5p-BMSCS-exsome protect cardiomyocytes by inhibiting the expression of Bax	Wu et al. (2021)
BMSCs	Mouse	Overexpression miR-132	miR-132	HUVEC	MI mouse	MiR-132-BMSC-Exos promote MI mice cardiac function by promoting the proliferation of HUVEC	Ma et al. (2018)
BMSCs	Mouse	Overexpression miR-126	miR-126	H/R treatment HUVEC		MiR-126-BMSC-Exos promote HUVEC to proliferate and migrate by improving PI3K/Akt/eNOS signaling pathway	Pan et al. (2019b)
BMSCs	Rat	Overexpression miR-338	miR-338	H2O2 treatment H9C2	MI mouse	MiR-338-BMSC-Exos can directly downregulate MAPK and inhibit JNK, thereby protecting cardiac function of infarction rats	Fu et al. (2020)
BMSCs	Rat	Overexpression miR-301	miR-301		MI rat	MiR-301-BMSC-Exos decrease the ratio of autophagy marker LC3-II/LC3-I in cardiomyocytes and upregulates P62, thereby protects cardiac function and cardiomyocytes in MI rats with myocardial infarction	Li et al. (2019)
BMSCs	Mouse	Overexpression miR-185	miR-185	HEK293 cell	MI mouse	Upregulation of miR-185 expression in BMSC-Exos can directly inhibit SOCS2 in ischemic site, thereby protecting the cardiac function of MI mice	Li et al. (2020b)
BMSCs	Rat	Overexpression miR-96	miR-96	H9C2 cell	Doxorubicin-Induced myocardial rat	Mir-96 protects Dox-induced rat by inhibition of Rac1/NF-κB	Lei et al. (2021)
BMSCs	Mouse	Overexpression miR-129-5p	miR-129-5p	OGD treatment HL-1 cell	MI mouse	MiR-129-5p in BMSC-Exos protected OGD-treated HL-1 cell by inhibiting TRAF3 expression. The study of Shuo Upregulating miR-129-5p in BMSCS-Exo could directly inhibit HMGB1 at the site of cardiac injury in mice, thereby protecting MI mouse cardiac function	Wang et al. (2022), Yan et al. (2022)
BMSCs	Rat	Overexpression miR-183-5p	miR-183-5p	H/R treatment NRCM	I/R rat	Upregulation of miR-183-5p expression in BMSCS-Exos can directly inhibit FOXO1 in I/R rat injury site, thereby protecting the cardiac function of I/R rat	Mao et al. (2022)
BMSCs	Mouse	Overexpression miR-182-5p	miR-182-5p	I/R treatment NRCM	H/R mouse	Upregulating miR-182-5p expression in BMSC-Exos can directly inhibit the expression of GSDMD at cardiac injury area, thereby protecting the cardiac function of H/R mice	Yue et al. (2022)
alloUMSCs		Knockout beta 2 microglobulin	miRNA-24	H9C2	MI rat	MiR-24 in B2M-UMSC-Exo protects cardiac function in MI rat by directly inhibiting Bim expression	Shao et al. (2020)

### 4.1 NC-RNA in MSC-Exos is changed by drugs and chemicals

**FIGURE 3:**
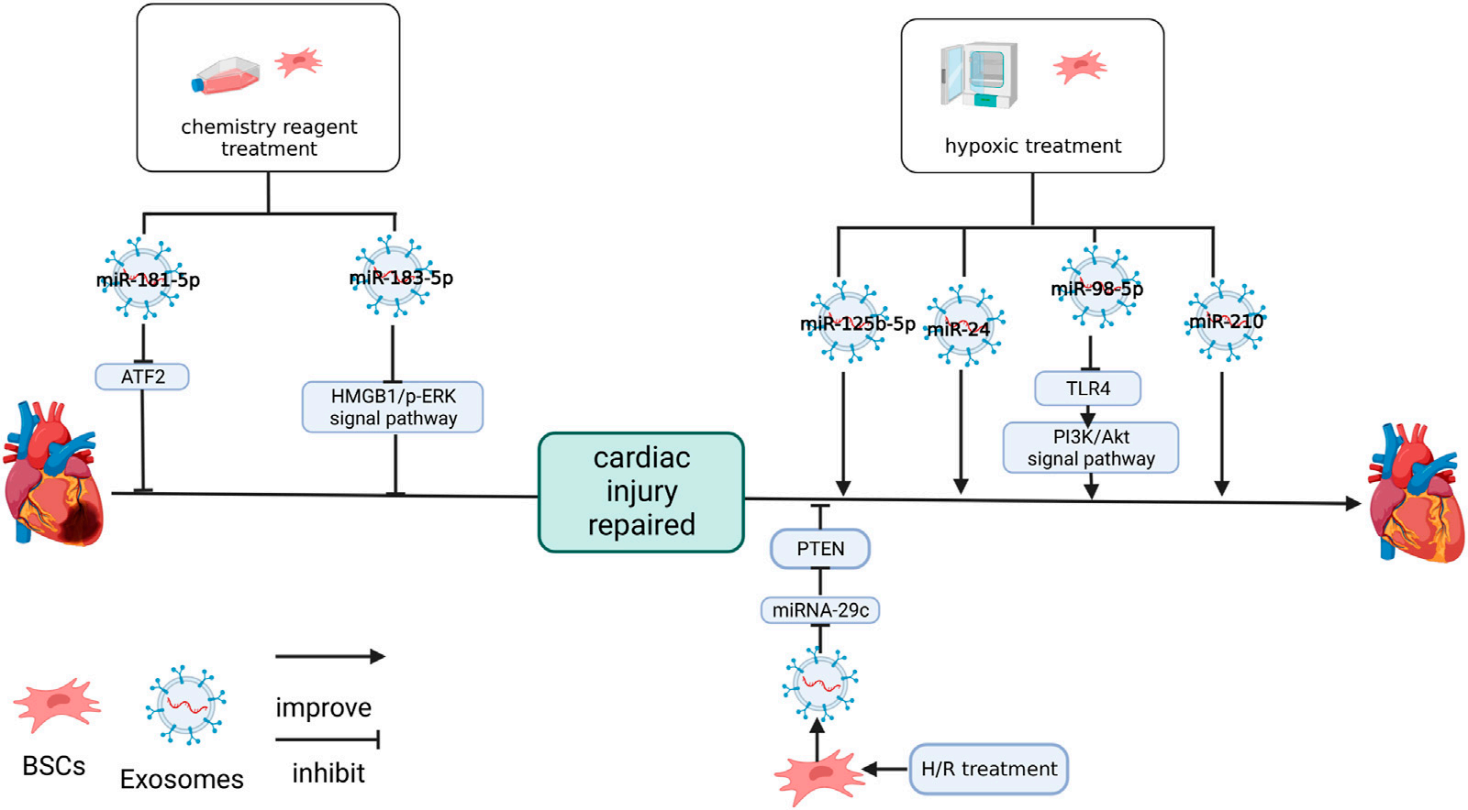
Effects of culture methods on MSC-Exos and NC-RNA. LPS upregulated miR-181-5p in BMSC-Exos. Hemin upregulated miR-183-5p in MSC-Exos. Both miR-181-5p and miR-183-5p protect cardiomyocytes by inhibiting the target genes. Hypotreatment upregulated miR-125b-5p (Zhu et al., 2018b), miR-24 (Zhang et al., 2019), miR-98-5p (Zhang et al., 2021) and miR-210 (Zhu et al., 2018a) in BMSC-Exos. miR-98-5p protects cardiac function by inhibiting the target gene, improving PI3K/AKT signaling pathway (Zhang et al., 2021). H/R treatment inhibited miR-29c in BMSC-Exos and impaired the protective effect on the heart of BMSC-Exos (Li et al., 2020a). The figure was created with BioRender.com.

The expression of NC-RNA in MSC-Exos may be changed by treatment with drugs and chemicals (Figure 3). Liu et al. (2020) found that exosomes differentiated from lipopolysaccharide (LPS)-stimulated MSCs (LPS-EXO) enhanced the resistance of H9C2 cells to H2O2 and upregulated miR-181-5p in H9C2 cells treated with H2O2 and the overexpression of miR-181-5p significantly downregulated apoptosis-related factors (TNF-α and IL-1β) at the protein level, decreased reactive oxygen species (ROS) and upregulated SOD1 and SOD2 in H9C2 cells. The target site of miR-181-5p was predicted to be located in ATF2. Treatment with the miR-181A-5p mimic can downregulated the expression of ATF2 in H9C2 cells, but the mutation of ATF2-predicted site interrupted this association (Liu et al., 2020).

In an earlier study, pretreating with hemin improved the MSC protection effect after MI (Deng et al., 2020). In a further study, it was shown that miR-183-5p in hemin-treated MSC-Exos (Hemin-MSC-Exos) was significantly upregulated. Additionally, it was revealed that the knockdown of miR-183-5p inhibited the protection of hemin-MSC-Exos in neonatal mouse cardiomyocytes (NMCM), reducing myocardial mitochondrial fission and inhibiting the expression of HMGB1, P-DRP1/Drp1 and P-ERK/ERK in NMCM after hemin-MSC-Exos treatment (Zheng et al., 2021). Specific downregulation of miR-183-5p inhibits this effect. At the same time, the miR-183-5p mimics downregulated HMGB1 in NMCM. The mutation of the miR-183-5p predicted target on HMGB1 will sever this association. Knockdown of miR-183-5p reduces the cardiac protection of hemin-MSC-Exos (Zheng et al., 2021).

### 4.2 NC-RNA in hypoxia-induced MSC-Exos

The oxygen content of the culture environment can also affect MSCs and MSC-Exos (Figure 3). In 2008, Rosova et al. (2008) found that hypoxia-induced MCSs (after 24 h of hypoxic culture) increased the expression of cMet, the primary receptor for hepatocyte growth factor expression, in ischemic areas and promoted the early restoration of blood flow. This phenomenon of MSCs may be related to the environment in which they are grown *in vivo*. When MSCs are cultured *in vitro*, they are usually under normoxic conditions (21% oxygen), but this oxygen level cannot be reached in physiological conditions, such as bone marrow or other environments (Rosova et al., 2008). This may suggest that when applying MSC-Exos to clinical practice, we should consider matching their physiological environment as much as possible to improve the effect of MSC-Exos.

The MSCs and the exosomes secreted by them were affected by a hypoxic environment. After the treatment of hypoxia-conditioned MSC-Exos (Hypo-Exo), six miRNAs (e.g., miR-5112, miR-711, miR-125b, miR-92a, mIR-7025, and miR-7045) were significantly upregulated in the MI tissue. miR-690, miR-6240, miR-125a, miR-3620, miR-6906 and miR-455 were also upregulated, but there were no significant difference (Zhu et al., 2018b). When miR-125b-5p decreased, the protective ability of Hypo-Exo on the hearts of mice with MI was significantly reduced and the recovery of Hypo-Exo on cardiac fibrosis and cardiac function injury was inhibited. Knockdown of miR-125b expression in Hypo-Exo can promote the expression of apoptosis markers (p53 and BAK1) in H9C2 cell after hypoxia treatment (Zhu et al., 2018b).

miR-24 was significantly expressed in the hypoxia-treated MSC-Exos and miR-24 was downregulated in the MI area of mice. Hypo-Exo treatment upregulates the expression of miR-24, reduces infarct size, recovers cardiac function and inhibits apoptosis (Zhang et al., 2019). These changes may be related to the downregulation of apoptosis-related molecules, such as Bax, cleaved caspase-3 and caspase-3. These effect of hypo-Exo treatment were abolished *in vitro* treated with the miR-24 inhibitor (Zhang et al., 2019).

Zhang et al. (2021) detected that miR-98-5p in Hypo-Exo was upregulated and inhibiting miR-98-5p reversed the effect of Hypo-Exo on the I/R rats, overexpression of miR-98-5p had a protective effect similar to Hypo-Exo in the I/R rats and overexpression of TLR4 inhibits the effect of miR-98-5p. In the cardiomyocytes of I/R rats, TLR4 was upregulated, miR-98-3p was downregulated, and p-Akt was downregulated, hypo-Exo can inhibit this trend, and overexpression of miR-98-5p can play the same role (Zhang et al., 2021). Additionally, miR-98-5p inhibited TLR4 expression in the cardiomyocytes, and mutation of the predicted site on TLR4 severed this link (Zhang et al., 2021).

Inhibitor-specific interference of miR-210 can inhibit the effect of Hypo-Exo on angiogenesis and anti-apoptosis of cardiomyocytes. The expression of miR-210 in Hypo-Exo treated with the inhibitor of nSMasw 2, which is sensitive to oxygen content and regulates exosome secretion, was downregulated, but the expression of miR-210 was unaffected in MSC-Exo which cultured in typical oxygen environments and inhibition of HIF-1α, a regulator of hypoxia response, inhibited the expression of nSMasw2 (Zhu et al., 2018a). These results suggest that nSMase2 is a hypoxic sensitive gene regulated by HIF-1 and that the repair function of Hypo-Exo may be regulated by factors related to oxidative stress response (Zhu et al., 2018a).

Interestingly, although hypoxic environments promote the repair of exosomes, it has been shown in studies that the H/R environment inhibits their protective effects. Li et al. (2020a) found that H/R inhibited the cardiac protective function of MSC-Exos and downregulated miR-29c while overexpressing miR-29c in I/R mice improved cardiac function and reduced the scar area. The inhibition of miR-29c expression in H/R primary rat cardiomyocytes upregulated autophagy factors LC3 and P62 *in vitro*. This was shown by the detection of target sites that miR-29c acted on PTEN and played a negative regulatory role (Li et al., 2020a).

### 4.3 Exosomes that alter the expression of specific genes

**FIGURE 4:**
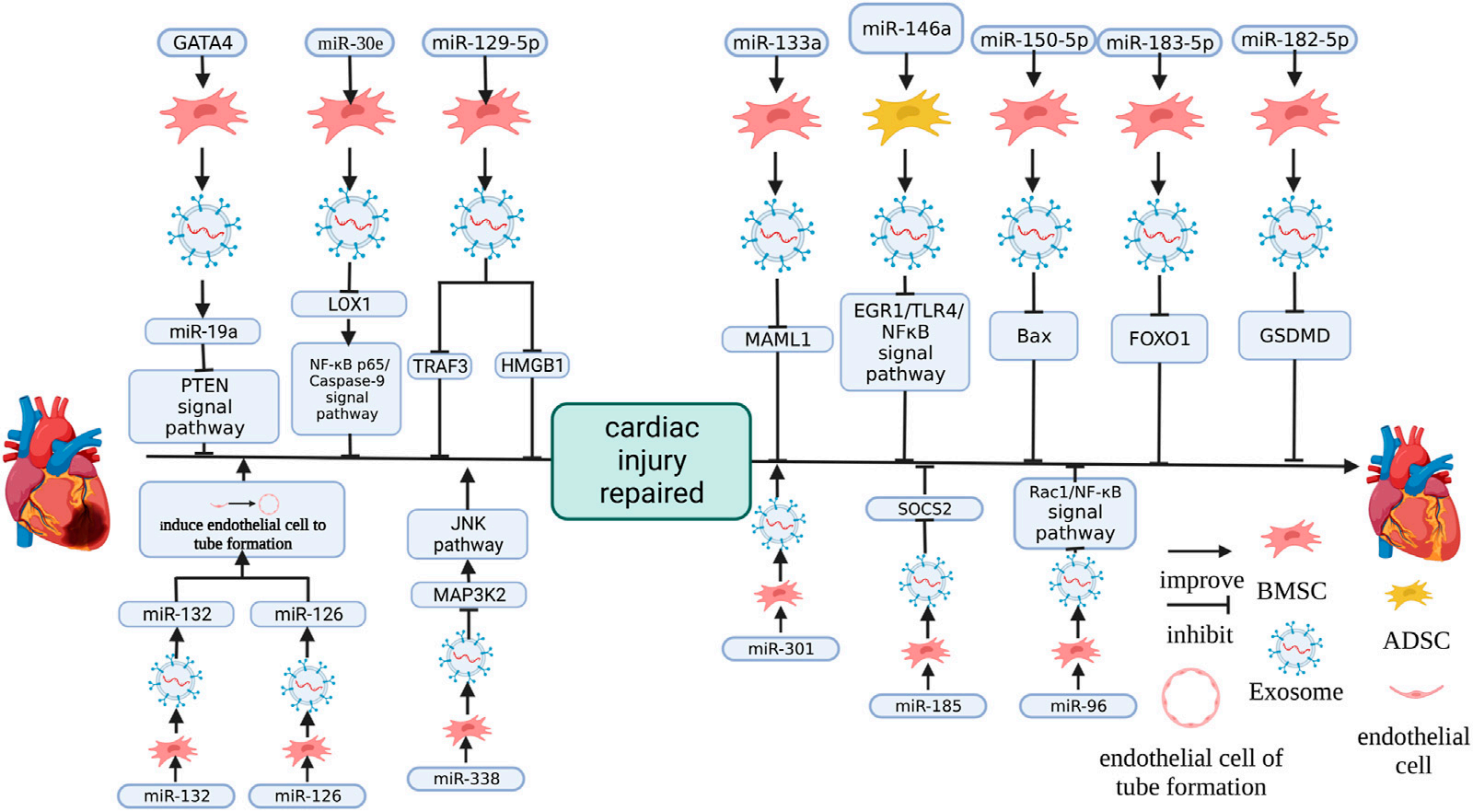
The effect of changing the expression of MSC-Exos miRNA on therapeutic effects. miR-126 (Pan et al., 2019b) and miR-132 (Ma et al., 2018) promoted cardiac function by promoting endothelial cells to form blood vessels. miR-338 (Fu et al., 2020), miR-19a (Yu et al., 2015), miR-30e (Pu et al., 2021), miR-129-5p (Wang et al., 2022; Yan et al., 2022), miR-133a (Li et al., 2021b), miR-146a (Pan et al., 2019a), miR-150-5p (Wu et al., 2021), miR-183-5p (Mao et al., 2022), miR-182-5p (Yue et al., 2022), miR-185 (Li et al., 2020b) and miR-96 (Lei et al., 2021) promote cardiac function recovery by inhibiting the target gene. miR-301 (Li et al., 2019) promoted the recovery of cardiac function by inhibiting apoptosis. The figure was created with BioRender.com.

Directly changing the expression of signaling molecules in MSC-Exos can change the therapeutic effect of MSC-Exos (Figure 4). Yu et al. (2015) found that GATA-4 overexpression of BMSC-Exos (Exo^GATA-4^) enhanced the protective function on NRCM cultured in hypoxia. I/R rats treated with Exo^GATA-4^ maintained a high survival rate, significantly reduced the proportion of apoptosis and myocardial fibrosis and recovered cardiac function in the MI area (Yu et al., 2015). It was shown in a component analysis of Exo^GATA-4^ that miR-19a and miR-451 were significantly increased and the protective effect of Exo^GATA-4^ was eliminated by the knockdown of miR-19a, this effect includes the inhibiton of Exo^GATA-4^ on autophagy and lactate dehydrogenase (LDH) release of CM (Yu et al., 2015). The predicted targets of miR-19a were PTEN and BCL and Exo^GATA-4^ significantly altered the expression of PTEN and its signaling pathway members p-Akt, BIM and P-ERK in CM (Yu et al., 2015).

Similarly, NC-RNA overexpression, which promotes damage repair, had a similar effect. In HF patients, miR-30e was significantly downregulated and it was also significantly downregulated in rats with HF (Marfella et al., 2013; Pu et al., 2021). The overexpression of miR-30e in BMSC-Exos (miR-30e-BMSC-Exos) significantly improved cardiac function, recovered myocardial injury, reduced infarct size, pathological injury score of myocardial tissue, the expression level of fibrosis-related factor α-SMA, cell apoptosis and promoted angiogenesis and the inhibition of inflammation induced by a heart attack on mice with MI and miR-30e-BMSC-Exos enhanced the activity, decreasing OGD and inhibiting OGD-induced apoptosis, cytotoxicity and further reduced the expression of fibrosis markers α-SMA and FN-1 of H9C2 cells (Pu et al., 2021). The target site of miR-30e was shown by the target site detection to be located in the LOX1 gene and the overexpression of LOX1 inhibited the enhancement of miR-30e-BMSC-Exos activity on H9C2 cells, increased the proportion of apoptotic cells and enhanced OGD-induced cytotoxicity and fibrosis (Pu et al., 2021). miR-30e-BMSC-Exos blocked NF-κB p65/caspase-9 signaling pathway and further overexpressing LOX1 would restore NF-κB p65/caspase-9 signaling pathway in OGD-H9C2 cells (Pu et al., 2021).

In rats, overexpression of miR-133a BMSC-Exos ameliorates Coxsackie B3 virus-induced viral myocarditis (VMC), which causes shaggy hair, dyspnea, poor diet, body weight loss, improved LVPW and LVID and decreased LVEF and FS, and the inhibition of miR-133a expression limited this effect (Li et al., 2021b). The target of miR-133a was located on MAML1, and the upregulation of miR-133a inhibited the expression of MAML1 (Li et al., 2021b). After si-MAML1 treatment, VMC rats gained weight, improved cardiac function, attenuated myocardial histopathology and fibrosis and inhibited the serum inflammatory response and apoptosis of cardiomyocytes (Li et al., 2021b).

In previous studies, miR-146a has been shown to regulate immune responses and reduce the degree of fibrosis in damaged organs in previous studies (Morishita et al., 2015; Lee et al., 2016). Adipose-derived stem cell exosomes overexpressing miR-146a (miR-146a-ADSC-Exo) reduce the infarct size of rats with MI, simultaneously decreased the degree of cardiomyocyte apoptosis and cardiac tissue fibrosis, decreased IL-6 (A), TNF-α and IL-1β (B) in the serum, downregulated the EGR1 and TLR4-related factors of EGR1/TLR4/NF-κB and improved the phosphorylation of NF-κB p65 (Pan et al., 2019a). *In vitro*, EGR1, which were co-cultured with miR-146a, was significantly decreased, apoptosis was significantly inhibited, and TNF-α, IL-1β and IL-6 were downregulated after miR-146a treatment (Pan et al., 2019a). It has been shown in WB results that caspase-3, α-SMA, fibrotropic protein collagen I and Bax were significantly downregulated, and the phosphorylation of NFκB p65 was increased and the above effects were reversed by transfection of the EGR1 overexpression vector *in vitro* (Pan et al., 2019a).

It was confirmed in an early study that miR-150-5p inhibited hypoxia-induced cardiomyocyte apoptosis caused by regulating B-cell cancer-associated X (Bax) (Scrutinio et al., 2017). BMSC-Exos overexpressing miR-150-5p (miR-150-5p-BMSC-Exos) significantly increased the cardiac function of mice with MI, the orderly arrangement of myocardial fibre, inhibited cardiac apoptosis and infiltration of inflammatory cells between myocardium, reduced the gap between myocardial cells was, and the expression of Bax (Wu et al., 2021).

In acute MI (AMI), vascular regeneration is required to reduce injury due to cardiac ischemia (Ma et al., 2018). Overexpressing miR-132 BMSC-Exos (miR-132-BMSC-Exos) co-cultured with HUVEC *in vitro* promotes their proliferation to form a tubular structure (Ma et al., 2018). After mice with MI were treated with miR-132-BMSC-Exos, cardiac function was significantly improved and capillary density in the injury place was increased (Ma et al., 2018).

miR-126 in MSCs can enhance cell survival ability, enhance the MSC therapeutic effect of transplantation on functional angiogenesis of the ischemic myocardium and promote the secretion of angiogenic factor (Huang et al., 2013a; Huang et al., 2013b). Overexpressing miR-126 BMSC-Exos (miR-126-BMSC-Exos) significantly increased the proliferating and migrating of hypoxia/reoxygenation (H/R) in HUVEC and significantly increased the tubular structure formed by HUVEC. This indicates that miR-126 in BMSC-Exos is essential, and this function has been confirmed to be realised through PI3K/Akt/eNOS (Pan et al., 2019b).

miR-338 in MSC-Exos has been confirmed to regulate apoptosis, the proliferation of colliculus cells and the growth of neural cells and miR-338 also plays a vital role in life activities, including tumour formation, cardiomyocyte autophagy, endothelial cell injury, etc. (Xin et al., 2012; Zhao et al., 2019b; Song et al., 2021; Fu et al., 2020) found that BMSC-Exos overexpressing miR-338 (miR-338-BMSC-Exos) improved the resistance of H9C2 cells to H2O2-induced injury, reduced apoptosis marker Bax and increased apoptosis antagonist Bcl-2, but an inhibitor of miR-338 reversed this phenomenon. The target gene of miR-338 is MAP3K2, a member of the MAPK signaling pathway (Fu et al., 2020). Overexpression of miR-338 reduced MAP3K2 and JNK, another member of the MAPK signaling pathway, in H9C2 cells at the protein level, and miR-338 inhibitors can reverse this result (Fu et al., 2020).

Li et al. (2019) showed that MSC-Exos overexpressing miR-301 significantly improved cardiac function, decreased the size of the cardiac infarction, the autophagy marker LC3-II/LC3-I ratio and reduced fibrosis and increased p62 in rats with MI.

Li et al. (2020b) found that overexpressing miR-185 promoted BMSC-Exos protective effects on the cardiac function of mice with MI and reduced the fibrosis, apoptosis of cardiomyocytes caused by MI and inhibited the expression of *suppressor of cytokine signaling 2 (SOCS2)* is a factor with a negative effect on I/R injury (Sheng et al., 2017). After overexpressing *SOCS2* and miR-185, cardiac function was significantly weakened and the degree of fibrosis was deepened, suggesting that miR-185 in exosomes protected the heart of MI by downregulating the target *SOSC2*.

BMSCs overexpressing miR-96 promoted miR-96 in BMSC-Exos, promoted BMSC-Exos protection on the cardiac function of DOX-induced rats, reduced free radicals and inhibited the toxicity of antioxidants and inhibited the toxicity of DOX, inflammatory reactions and fibrosis. This is achieved by miR-96 inhibiting the Rac1/NF-κB signaling pathway (Lei et al., 2021).

BMSC-Exos upregulating miR-129-5p in OGD-HL-1 and inhibiting miR-129-5p expression had a weak BMSC-Exos effect on OGD-HL-1 and overexpression of TRAF3 reverses the BMSC-Exos effect on OGD-HL-1 and this effect is achieved by activating NF-κB signaling (Yan et al., 2022). Overexpression of miR-129-5p inhibited TRAF3, and the mutation of TRAF3 at the predicted site interrupted this association (Yan et al., 2022). It was shown in another study that overexpressing miR-129-5p enhanced the BMSC-Exos effect on mice with MI by limiting HMGB1 expression in cardiomyocytes (Wang et al., 2022). Interestingly, previous research mainly emphasized that MSC-Exos was a suitable carrier of miRNA and carried miRNA into recipient cells. Still, the miR-129-5p mimic on BMSCs upregulated miR-129-5p and downregulated HMGB1 in BMSCs (Wang et al., 2022).

Mao et al. (2022) found that overexpressing miR-183-5p in BMSC-Exos (miR-183-5p-BMSC-Exos) had significant protection on I/R rats, inhibited the weakening of heart function and decreased MI area. This was caused by the direct inhibition of FOXO1 expression, and the upregulation of miR-183-5p downregulated FOXO1 expression and mutated the predicted target site on FOXO1, severing this link (Mao et al., 2022).

The expression of GSDMD was enriched in the I/R cardiac tissue of mice and GSDMD was the miR-182-5p target gene. Upregulating the expression of GSDMD in NMCM promoted pyroptosis and H/R cardiomyocyte injury (Yue et al., 2022). MSCs incubated with the miR-182-5p mimic can deliver miR-182-5p to I/R NMCM through MSC-Exos. The expression of GSDMD in cardiomyocytes was downregulated (Yue et al., 2022).

Knockout of a specific gene in MSCs caused changes in miRNA expression in MSC-Exos. Shao et al. (2020) discovered that *beta-2 microglobulin* knockout allogeneic human umbilical MSCs exosomes (alloUMSC-Exos^B2M−^) effectively protected cardiac function and reduced cardiac fibrosis and miR-24 was significantly increased in alloUMSC-Exos^B2M−^ and mice with MI treated with alloUMSC-Exos^B2M−^. It was shown in bioinformatics analysis that Bim was the miR-24 target gene and upregulation of miR-24 inhibited Bim expression, which was eliminated by a mutation of the Bim 3’UTR-predicted target site. The miR-24 inhibitor eliminated the therapeutic effect of alloUMSC-Exos^B2M−^ on mice with MI, reduced their cardiac function and upregulated the Bim expression and further the overexpression of miR-24 restored the protective effect of alloUMSC-Exos^B2M−^ on mice with MI (Shao et al., 2020).

## 5 Cardiac protective effect of lncRNAs in MSC-Exos

**FIGURE 5:**
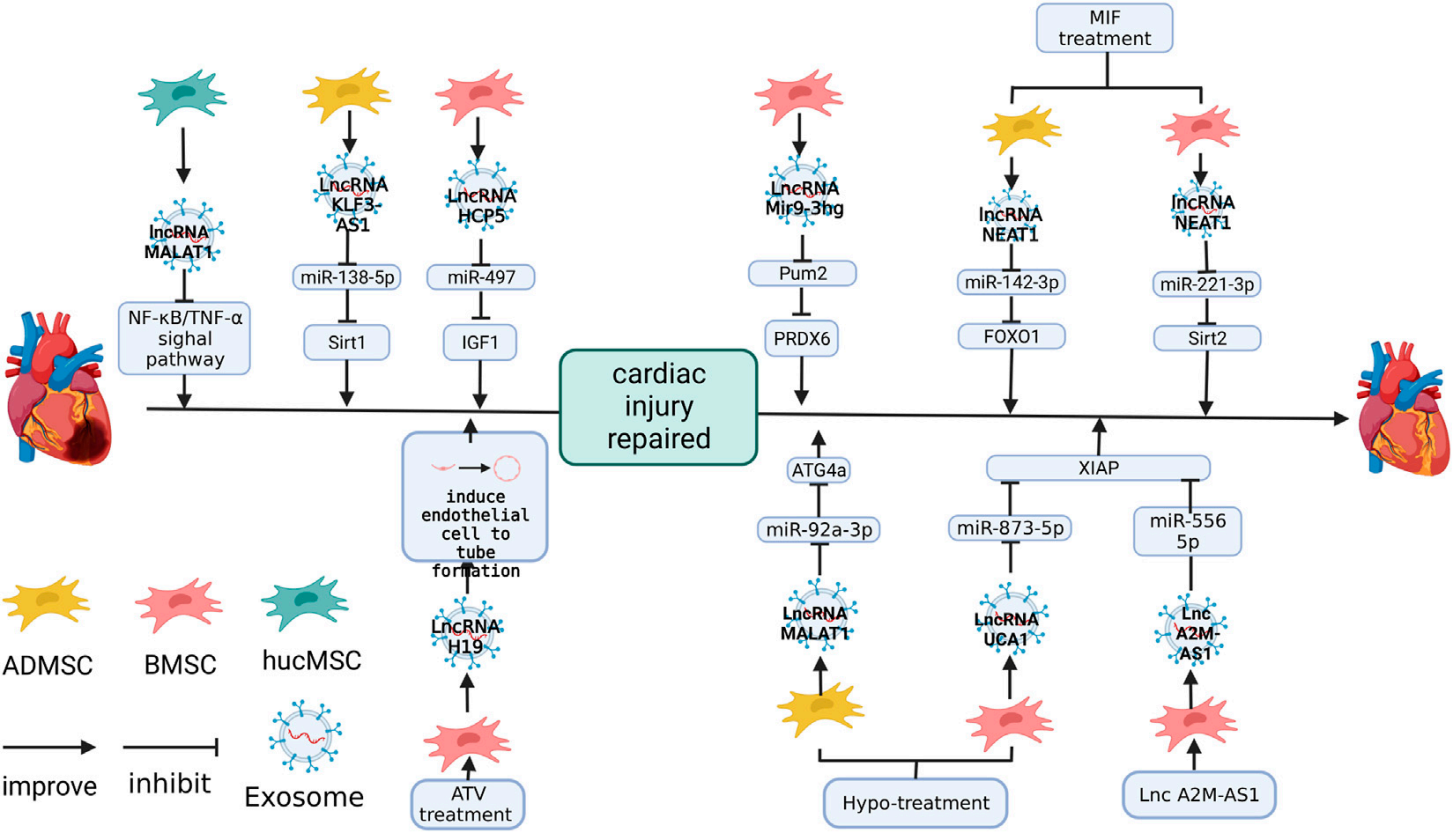
The role of lncRNAs in MSC-Exos in the repair of cardiac injury. lncRNA-H19 plays a role in promoting endothelial cell angiogenesis (Huang et al., 2020). lncRNA-MALAT1 (Xia et al., 2020), KLF3-AS1 (Mao et al., 2019), HCP5(Li et al., 2021a), NEAT1 (Chen et al., 2020), UCA1 (Sun et al., 2020) and Mir9-3 (Zhang et al., 2022) protect cardiac function by inhibiting the expression of target genes. The figure was created with BioRender.com.

NC-RNAs, which are over 200 nt, are called lncRNAs. It was confirmed in early studies that lncRNA is closely related to cancer and aging (Iyer et al., 2015; Marttila et al., 2020). MSC-Exos also contains many lncRNAs that are essential in protecting the heart. Here, we discuss the effect of lncRNAs in MSC-Exos in the repair of cardiac injury and related mechanisms. Additionally, we provided a summary in Table 3 and Figure 5.

**TABLE 3:** Role of lncRNA in MSC-Exos-induced cardiac injury repair and its related mechanisms.

Source of exosome	LncRNA	*In vitro*	*In vivo*	Mechanism	References
hucMSCs	lncRNA MALAT1	H_2_O_2 treatment H9C2_	D-gal treatment mouse	LncRNA MALAT1 in hucMSC-Exos protect cardiac function in D-Gal-induced senescent mice by inhibiting NF-κB/TNF-α signaling pathway	Zhu et al. (2019)
hMSCs	LncRNA KLF3-AS1	Hypo-H9C2	MI rat	LncRNA KLF3-AS1 in hMSC-Exos protect cardiac function of MI rats by directly inhibiting miR-138-5p in cardiomyocytes and upregulating Sirt1	Mao et al. (2019)
hBMSCs	LncRNA HCP5	H/R treatment human cardiomyocytes	I/R rat	LncRNA HCP5 in hBMSC-Exos protect cardiomyocytes by directly inhibiting miR-497 in cardiomyocytes and activating IGF1/PI3K/AKT signaling pathway	Li et al. (2021a)
MIF-ADMSCS	LncRNA NEAT1	H2O2 treatment Human-induced pluripotent stem cell (hiPSC) derive cardiomyocytes		Pretreatment with MIF upregulate LncRNA NEAT1 in ADMSC-Exos, and LncRNA-NEAT1 upregulate FOXO1 and inhibit the apoptosis by directly downregulating miR-142-3p in cardiomyocytes	Chen et al. (2020)
MIF-mice BMSCs	LncRNA NEAT1	Dox-NMCM	Dox-induce mouse	MIF can upregulate lncrNA NEAT1 in BMSC-Exos directly inhibit miR-221-3p in cardiomyocytes, thereby upregulating Sirt2 in cardiomyocytes and protecting cardiomyocytes	Zhuang et al. (2020)
ATV treatment BMSCs	lncRNA H19	H/SD treatment H9C2 and HUVEC	MI rat	Downregulating LncRNA H19 expression in BMSC-Exos^ATV^ attenuates the protective effect of BMSC-Exos^ATV^ on cardiomyocytes	Huang et al. (2020)
Hypo-BMSCs	LncRNA UCA1	H/SD treatment H9C2	MI rat	Hypoxia leads to improve lncrNA UCA1 expression in BMSCS, which directly downregulates miR-873-5p in cardiomyocytes and upregulate XIAP, thereby protecting cardiomyocytes	Sun et al. (2020)
Hypo-human adMSCs	LncRNA MALAT1	Dox treatment Human-induced pluripotent stem cell–derived cardiomyocytes		Hypoxia leads to upregulate LncrNA MALAT1 expression in ADMSC-Exos, directly downregulate miR-92A-3p and upregulate ATG4a in cardiomyocytes, and downregulate the senescence related genes p53 and p21 to protect cardiomyocytes	Xia et al. (2020)
Overexpressing Lnc A2M-AS1 in hBMSCs	Lnc A2M-AS1	H/R treatment AC16		Human BMSC-Exos overexpressing Lnc A2M-AS1 can upregulate XIAP by inhibiting the expression of miR-556-5p, thereby preventing cardiomyocytes apoptosis caused by H/R treatment	Yu et al. (2022)
Mouse BMSCs	lncRNA Mir9-3hg	H/R treatment HL-1	I/R mouse	The LncRNA Mir9-3hg in BMSC-Exos inhibit cardiomyocytes iron death through Pum2/PRDX6, thereby protecting cardiomyocytes and improving cardiac function in H/R mice	Zhang et al. (2022)

Zhu et al. (2019) found that lncRNA-MALAT1 in the heart is lost with age. At the same time, the expression of lncRNA-MALAT1 in hucMSC-Exos was abundant and cardiac function has a significant recovery in D-galactose (D-GAL) induced aged mice after hucMSC-Exos treatment, but silencing lncRNA-MALAT1 in hucMSC-Exos decreased its protective ability against cardiac function in D-GAL-induced aged mice. hucMSC-Exos upregulated the expression of anti-aging factor TERT and downregulated aging factor p21, inflammatory factors TNF-α and NF-κB P-p65, while siMALAT1 inhibited this effect (Zhu et al., 2019).

Overexpression of lncRNA KLF3-AS1 in hMSC-Exos (human mesenchymal stem cells) significantly improved the cardiac function of rats with MI, inhibited the infiltration of inflammatory cells, pro-inflammatory cytokines IL-18 and IL-1β, and reduced the apoptosis caused by hypoxia and inhibiting lncRNA KLF3-AS1 in hMSC-Exos led to the opposite effect (Mao et al., 2019). The software predicted that lncRNA KLF3-AS1 regulated Sirt1 through miR-138-5p and inhibited miR-138-5p, which upregulated both lncRNA KLF3-AS1 and Sirt1 (Mao et al., 2019). The overexpression of KLF3-AS1 downregulated miR-138-5p and upregulated Sirt1and a mutation of the predicted site invalidated this association (Mao et al., 2019).

Li et al. (2021a) found that lncRNA HCP5 in BMSC-Exos inhibited cardiomyocyte apoptosis and the overexpression of HCP5 in BMSC-Exos improved the viability of cardiomyocytes, downregulated miR-497, and the knockdown of HCP5 upregulated miR-497. Mutating the predicted target site of HCP5 and miR-497 stopped this connection, and the miR-497 mimic significantly attenuated the expression of IGF1 (Li et al., 2021a). It was shown in a study on H/R cardiomyocytes that HCP5 in BMSC-Exos inhibited apoptosis by miR-497/IGF1 (Li et al., 2021a).

The change in the external environment changed the expression of miRNA and the expression of lncRNA in MSC-Exos. Chen et al. (2020) found that co-culture of *macrophage migration inhibitory factor* (*MIF*) significantly improved the protective effect of ADMSC-Exos (ADMSC-Exos^MIF^) on hiPSC-derived cardiomyocytes in an H2O2 culture environment. *MIF* affected the expression of lncRNA in ADMSC-Exos, and lncRNA NEAT1 was significantly upregulated in ADMSC-Exos^MIF^. Reducing lncRNA NEAT1 by siRNA deprived the protective effect of ADMSC-Exos^MIF^ on cardiomyocytes (Chen et al., 2020). miR-142-3p was regulated by lncRNA NEAT1 and the overexpression of miR-142-3p increased the apoptosis rate of cardiomyocytes and enhanced the activities of caspase eight and caspase 3/7 even in the environment of ADMSC-Exos^MIF^ incubation (Chen et al., 2020). miR-142-3p inhibited FOXO1, nullifying the inhibitory effect of ADMSC-Exos^MIF^ on ROS generation, MDA, 4-HNE activities and superoxide dismutase activation in H2O2-treated cardiomyocytes (Chen et al., 2020).

miR-142-3p is not the only target gene for lncRNA NEAT1. It was shown in the study of Zhuang et al. (2020) on lncRNA NEAT1 in BMSC-Exos^MIF^ that its inhibition upregulated miR-221-3p in the heart of mice treated with DOX chemotherapy, and predicted target site mutations of lncRNA NEAT1 and miR-221-3p nullified this effect. Transduction of the miR-221-3p mimic into DOX-treated mice impaired the protection of BMSC-Exos^MIF^ on cardiac function in DOX-treated mice and also led to the upregulation of the transcription levels of cell senescence-related factors, such as p27 and p16, in the heart of DOX-induce-mice (Zhuang et al., 2020). It was also shown by Sa-β-gal staining result that cardiomyocytes of DOX-induce-mice had a higher degree of senescence, which was achieved by directly downregulating the expression of Sirt2 (Zhuang et al., 2020).

Huang et al. (2020) found that BMSC-Exos^ATV^ secreted from the BMSCs of rats treated with atorvastatin (ATV) significantly improved the formation, migration and survival of HUVECs *in vitro*. After direct injection into the heart of rats with MI, BMSC-Exos^ATV^ significantly improved cardiac function, reduced the degree of cardiac fibrosis, promoted the generation of blood vessels and the survival of cardiomyocytes and significantly reduced inflammatory factor TNF-A in cardiac (Huang et al., 2020). The expression of lncRNA H19 was enriched in BMSC-Exos^ATV^, and the silencing of lncRNA H19 significantly reduced the length of tubules formed by co-cultured HUVEC and impaired migration, cell survival and decreased cardiac function, increased the MI area *in vivo* (Huang et al., 2020).

Similarly, the expression of lncRNA in MSC-Exos cultured under hypoxia also changed. Sun et al. (2020) detected that lncRNA UCA1 was significantly upregulated in Hypo-BMSC-Exos and knockdown of lncRNA UCA1 in Hypo-BMSC-Exos reduced the protection of Hypo-BMSC-Exos in rats with MI, deepened the degree of cardiac fibrosis, increased the infarct area, and upregulated cleaved caspase-3 and Bax. lncRNA UCA1 downregulated miR-873-5p in H9C2 cells and further increased XIAP, and the overexpression of miR-873-5p in cardiomyocytes promoted the apoptosis of H/R H9C2(Sun et al., 2020). This suggested that UCA1/miR-873-5p/XIAP dingle was essential in protecting Hypo-BMSC-Exos against MI (Sun et al., 2020).

Xia et al. (2020) found that lncRNA MALAT1 in Hypo-ADMSC-Exos was significantly increased, and ADMSC-Exos overexpressing lncRNA MALAT1 reduced the damage to cardiomyocytes induced by DOX and silencing lncRNA MALAT1 inhibited this effect. The target gene of lncRNA MALAT1 was miR-92a-3p, knockdown lncRNA MALAT1 in ADMSC-Exos upregulated miR-92a-3p and this relationship ended after mutating the predicted target site and overexpression of miR-92a-3p eliminated the effect of ADMSC-Exos on DOX-induced cardiomyocytes. Additionally, ATG4a was negatively regulated by miR-92a-3p and inhibiting ATG4a expression by RNAi upregulated the senescence-related genes p53 and p21 in cardiomyocytes (Xia et al., 2020). This indicates that Hypo-ADMSC-Exos may regulate miR-92A-3p/ATG4a in cardiomyocytes through lncRNA MALAT1, thereby achieving antagonism against DOX (Xia et al., 2020).

MSC-Exos can also be the carrier of lncRNAs. lncA2M-AS1 significantly decreased in AMI patient blood and H/R-treated AC16 cells. Yu et al. (2022) found that overexpressing lncA2M-AS1 in human bone marrow MSC-Exos (lncA2M-AS1-hBMMSC-Exos) effectively upregulated lnc A2M-AS1 in H/R-treated AC16 cells and enhanced AC16 cells activity. Further studies showed that the direct target site of lncA2M AS1 was miR-556-5p and lncA2M AS1 plays a negative regulatory role in miR-556-5p, while mutation of the predicted site interrupts this association (Yu et al., 2022). The target site of miR-556-5p was predicted to be located at the 3’end of XIAP, while mutation of the XIAP 3’end interrupted the relationship between XIAP and miR-556-5p. Upregulating miR-556-5p in H/R-treated AC16 cells reversed the repair effect of lncA2M-AS1-hBMMSC-Exos, and downregulation of XIAP expression had the same effect (Yu et al., 2022).

In addition to their role in traditional apoptosis, it has been shown in a recent study that lncRNA in MSC-Exos also affects induced iron death. lncRNA Mir9-3hg was enrichment in mouse MSC-Exos, and lncRNA Mir9-3hg was upregulated in cardiac myocytes of H/R HL-1 mice treated with MSC-Exos (Zhang et al., 2022). It was shown that upregulating lncRNA MIR9-3Hg inhibited Pum2 in cardiomyocytes and then upregulated PRDX6, inhibiting the iron death of cardiomyocytes and this function of BMSC-Exos was demonstrated in I/R mice. BMSC-Exos was upregulated mir9-3Hg, PRDX6 and Gpx4, which was lost due to H/R, and downregulated Pum2 and ACSL4, enriched due to H/R (Zhang et al., 2022).

## 6 Conclusion and perspectives

In earlier studies, mesenchymal stem cells were seen as a substitute for damaged tissue. MSCs injected *in vivo* tend to liver, and host myocardial cells will promote MSCs apoptosis through paracrine, which makes it difficult to retain a large number of MSCs. Therefore, MSC-Exos is regarded as a new treatment scheme (Hu et al., 2018). Several experiments have shown that MSC-Exos can repair heart damage caused by many reasons. This article describes the effect of MSC-Exo on cardiovascular disease from the perspective of non-coding RNA.

Although MSC-Exos have been proved to be effective in reducing the damage caused by cardiovascular disease, the source of MSC-Exos is a controversial topic. The naming rules of MSCs are based on naming hematopoietic stem cells that develop into mesenchymas, and the components of MSC-Exos in different parts were very different. As a result, different types of MSC-Exos have significant differences in the therapeutic effect. For example, EnMSC-Exos is better than that of adMSC-Exos and BMMSC-Exos (Wang et al., 2017).

It has been confirmed in several studies that MSC-Exos are suitable carriers of NC-RNA. Overexpression of NC-RNA, which has been proven to affect wound healing, can improve the therapeutic repair effect of MSC-Exos on cardiac injuries. Such NC-RNA will restore cardiac function by inhibiting the expression of apoptotic factors, but will also affect the expression of such apoptotic factors in MSCs (Wang et al., 2022). Whether this will have different effects is unclear.

The role of MSC-Exos is to reduce the apoptosis of myocardial cells, inhibit the infiltration of inflammatory factors, promote the formation of blood vessels and reduce fibrosis. Most studies focused on activating proliferation related signaling pathways and inhibiting apoptosis related factors in cardiomyocytes and endothelial cells, while less attention was paid to the molecular mechanism of MSC-Exos on the immune system and fibroblasts. Recent studies have shown that non coding RNA in MSC-Exos inhibits mTOR in fibroblasts (Fan et al., 2022), while MSC-Exo activates mTOR related signaling pathways in cardiomyocytes (Wang et al., 2017). In addition to the traditional AKT/PI3K signaling pathway and the NF-κB related signaling pathway, recent studies have shown that non-coding RNA in BMSC-Exos inhibits iron death in cardiomyocytes through Pum2/PRDX6 single pathway. The above examples show that the effects of MSC-Exos on heart injury are far beyond the current understanding. Further understanding of these effects will help us to further understand MSC-Exo and propose further application strategies.
